# *Physarum*Spreader: A New Bio-Inspired Methodology for Identifying Influential Spreaders in Complex Networks

**DOI:** 10.1371/journal.pone.0145028

**Published:** 2015-12-18

**Authors:** Hongping Wang, Yajuan Zhang, Zili Zhang, Sankaran Mahadevan, Yong Deng

**Affiliations:** 1 School of Computer and Information Science, Southwest University, Chongqing, China; 2 School of Information Technology, Deakin University, Geelong, VIC, Australia; 3 Civil and Environmental Engineering Department, Vanderbilt University, Nashville, TN, United States of America; Wake Forest School of Medicine, UNITED STATES

## Abstract

Identifying influential spreaders in networks, which contributes to optimizing the use of available resources and efficient spreading of information, is of great theoretical significance and practical value. A random-walk-based algorithm *LeaderRank* has been shown as an effective and efficient method in recognizing leaders in social network, which even outperforms the well-known PageRank method. As LeaderRank is initially developed for binary directed networks, further extensions should be studied in weighted networks. In this paper, a generalized algorithm *Physarum*Spreader is proposed by combining LeaderRank with a positive feedback mechanism inspired from an amoeboid organism called *Physarum Polycephalum*. By taking edge weights into consideration and adding the positive feedback mechanism, *Physarum*Spreader is applicable in both directed and undirected networks with weights. By taking two real networks for examples, the effectiveness of the proposed method is demonstrated by comparing with other standard centrality measures.

## Introduction

Over the years, the study of graphs and networks have drawn increasing attention in a wide variety of scientific disciplines, such as biology, computer science, economics, mathematics and sociology. Meanwhile, network analysis, as a key tool to map and measure those network entities and their connections, has been well studied to provide a visual and mathematical view of networks. However, a major challenge it encounters is how to identify the most efficient spreaders for optimizing the use of available resources and ensuring efficient spread of information [1–5]. With different topology and relations, node centrality can be endowed with various meanings, such as influence [6], importance [7, 8], popularity [9, 10], controllability [11, 12] and spreading efficiency [1]. Studies on the importance of a node for spreading can be of great significance in controlling rumor, disease spreading and information flow in networks [13–16].

Ever since the idea of centrality was introduced, various centrality measures have been proposed to identify nodes which are more central than others [17–19]. Recently, Landherr et al. [20] conducted a critical review of five common centrality measures in social networks, including degree centrality [17], closeness centrality [17], betweenness centrality [17], eigenvector centrality [21] and Katz’s centrality [22]. *Degree*, the simplest centrality measure, is the number of edges that a node is connected to, which was firstly proposed by Freeman. However, a node with higher degree might not be in a position which can access resources quicker. In order to make up this drawback, a more sophisticated centrality measure *closeness* is developed, which was defined as the inverse total length from a node to all other nodes. Another important centrality measure is *betweenness*, which is calculated as the fraction of the times that a node lies on the shortest paths over the total number of the shortest paths. Besides, by introducing random walks, a revised betweenness centrality is proposed in [23], which counts the frequency of a node traversed by a random walk between two other nodes. And then, a random-walk-based centrality called *LeaderRank* [9] has been proposed, which can identify leaders in social networks better than the well-known *PageRank* algorithm. After that, Chen et al. [6] developed a semi-local centrality measure as a tradeoff between local degree centrality and other global but time-consuming measures. Ranking influential nodes can be seen as a multi-attribute decision making problem [24]. Due to the efficiency to combine different data [25–29], evidence theory is also widely used for identifying influential spreaders in complex networks [30, 31].

However, those measures described above are only suitable in binary networks. In many real networks, edges are with some form of attributes or weights, rather than simply either present or absent in a pair of nodes. If only binary networks are considered, ignoring the intrinsical weights attached to edges, plenty of valuable information has been lost and the analysis cannot be accurate and comprehensive. Thus, many researchers have turned their attention to centrality for weighted networks [32–36].

In 1991, Freeman et al. [37] introduced a new measure of centrality based on the concept of network flows, flow betweenness, which considered all the independent paths between all pairs of nodes in the network. Based on the degree centrality, Barrat et al. [38] proposed a measure for weighted networks, which is the sum of weights of edges that a node is connected to. Beyond that, Newman [32] and Brandes [39] have generalized the closeness and betweenness centrality for weighted networks by using Dijkstra’s algorithm [40] on computing the shortest paths. By taking both edge weights and the number of edges into consideration, a new generalization was proposed by Opsahl et al. [33], using a tuning parameter to balance the relative importance between those two parts. Later, Qi et al. [34] developed a Laplacian centrality method considering “intermediate” environmental information around a node.

In this paper, we proposed a generalized centrality metric, called *Physarum*Spreader, to identify nodes with high spreading performance in networks. *Physarum*Spreader is developed on the basis of a random-walk-based algorithm LeaderRank and a positive feedback mechanism inspired from an amoeboid organism, called *Physarum Polycephalum*. With the integration of the algorithm and mechanism, our *Physarum*Spreader is applicable in both directed and undirected networks with weights. It overcomes the shortcomings of LeaderRank which is only designed for binary network and does not work well for undirected networks. Furthermore, a susceptible-infected-removed (SIR) model is employed to examine the spreading performance of nodes identified by different centrality measures. With simulations on different networks and comparison with other centrality measures, it reveals that *Physarum*Spreader works well in identifying influential nodes with high spreading performance and good tolerance.

The rest of the paper is organized as follows. Section 2 begins with a brief introduction to LeaderRank algorithm and positive feedback mechanism of *Physarum Polycephalum* adopted in our method. Then, procedure of the proposed *Physarum*Spreader for identifying influential spreaders in networks is depicted in Section 3. And two applications in real networks are presented in section 4. What’s more, the spreading effectiveness and robustness are studied. Section 5 concludes the paper.

## 1 Basic Theory

### 1.1 LeaderRank for Identifying Leaders [9]

Given a directed network of *N* nodes and *M* edges, a *ground node* is then added by establishing bidirectional edges between it and all the other nodes, which assures the modified network as strongly connected. And the modified network consists of *N* + 1 nodes and *M* + 2*N* directed edges. Initially, each node in the network, except for the ground node, is assigned with one unit resource, while the ground node is assigned with no resource. And then each node evenly distributes its resource to neighbors along the outgoing edges. Next is to update resource distribution as summing up the resource each node derives from its incoming edges. This process of distribution and updating of resources continues until steady state is attained. The whole process can be described mathematically as follows.

Assuming *r*_*i*_(*t*) denotes the resource of node *i* at time *t*, the initial state (*t* = 0) of resource distribution can be represented as:

$$\begin{array}{c} \begin{array}{c} r_{g}\left(0\right)=0,\;where\;g\;is\;ground\;node\\ r_{i}\left(0\right)=1,\;where\;i\in \left\{1,2,\cdots ,N+1\right\}\backslash \left\{g\right\} \end{array} \end{array}$$ (1)

And each node can update its resource according to the following equation:

$$\begin{array}{c} r_{i}\left(t+1\right)=\sum_{j=1}^{N+1}\frac{a_{ji}}{k_{j}^{out}}r_{j}\left(t\right) \end{array}$$ (2)

where *a*_*ij*_ is the element of the corresponding (*N* + 1)-dimensional adjacency matrix, which equals 1 if there is a directed link from *j* to *i* and 0 otherwise, and $k_{j}^{out}$ is the out-degree of node *j*.

When the resource *r*_*i*_(*t*) at all nodes converges to a unique steady state at time *t*_*c*_, the resource at the ground nodes is then evenly distributed to all other nodes, and the final resource distribution on nodes *i* is:

$$\begin{array}{c} R_{i}=r_{i}\left(t_{c}\right)+\frac{r_{g}\left(t_{c}\right)}{N},\;where\;i\in \left\{1,2,\cdots ,N\right\} \end{array}$$ (3)

### 1.2 *Physarum* Model for Path Finding

*Physarum polycephalum*, as a large, single-celled amoeboid organism, can form a dynamic tubular network within the discovered food sources.

Recently, a large amoeboid organism, *Physarum polycephalum*, turned out to be capable of solving many graph theoretical problems [41–45], including finding the shortest path [46–48]. Furthermore, it has been shown experimentally that the network it generates is of high intelligence and performance in road-network [49] and great transport efficiency in vascular network [50], even comparable to or better than the Tokyo rail network [51]. During the process of its path finding and tube selection, *Physarum* will cut off those non-competing long tubes and reinforce shorter tubes. And, with the positive feedback mechanism among the tube length, the flux through tubes and the conductivity of tubes (tube width), shorter tubes result in larger flux; tube with a large flux grow (tube width increases); wider tubes leads to a further increase of flux as the resistance to the flow decreases in wider tubes. According to *Physarum*’s tubular network, each tube segment is regarded as an edge *e*_*ij*_ in graph and its two ends are denoted as nodes *i* and *j*, which the edge connects. For each tube segment, there are two critical attributes: one is the length of the tube *L*_*ij*_ and the other is its thickness, which is always represented as conductivity *D*_*ij*_. Based on the theory that thick, short tubes are typically the most effective for transportation, the mathematical model of *Physarum* includes two parts: flux through tubes and adaptation of conductivity according to its flux.

#### 1.2.1 Flux through Tubes [52]

As circulation is based on streaming through network of tubular channels, the flux of sol through the tubes *Q*_*ij*_ is approximately modeled as Poiseuille flow:

$$\begin{array}{c} Q_{ij}=\frac{D_{ij}}{L_{ij}}\left(p_{i}-p_{j}\right) \end{array}$$ (4)

where *p*_*i*_ is the pressure at node *i*.

By considering the balance of flux through each node, we have

$$\begin{array}{c} \sum_{i}\frac{D_{ij}}{L_{ij}}\left(p_{i}-p_{j}\right)=\left\{\begin{array}{c} -I_{0},\;\text{for}\;j=s,\\ +I_{0},\;\text{for}\;j=t,\\ \;\;\;0,\;\text{otherwise}. \end{array}\right. \end{array}$$ (5)

where *s* is the source node that the initial flux *I*_0_ flows out, while *t* is the sink node from which the flux flows in.

#### 1.2.2 Adaptation of Conductivity [52]

In order to model the positive feedback mechanism that tube widens with increasing flux and degenerates with decreasing flux, the conductivity *D*_*ij*_ is assumed to change over time according to the flux *Q*_*ij*_:

$$\begin{array}{c} \frac{d}{dt}D_{ij}=f\left(\left|Q_{ij}\right|\right)-\gamma D_{ij} \end{array}$$ (6)

where *γ* is a decay rate of the tube. *f* (*Q*) is an increasing function with *f* (0) = 0. More detailed description of *f* (*Q*) can be found in [53].

## 2 *Physarum*Spreader

LeaderRank is an efficient method for identifying influential leaders in opinion spreading and outperforms PageRank algorithm, the basis of the Google search engine, in ranking effectiveness and robustness against manipulations and noisy data. However, it is initially designed for binary networks, which is not suitable for weighted networks.

Consider that the resource distribution from each node to its neighbors in LeaderRank, is similar to the flowing of flux through tubes in *Physarum* model of path finding. Nevertheless, *Physarum* model is designed for finding the shortest paths in both binary and weighted networks, which is capable of handling edge weights. Thus, it is natural to consider that adoption of the positive feedback mechanism between conductivity and flux in *Physarum* model may be of great help in overcoming the weakness of LeaderRank in weighted networks.

The main mechanism behind the presented method is combining the positive feedback mechanism in *Physarum* model and resources distribution mechanism in LeaderRank. Specifically, each node proportionally distributes resources to its outlinks based on their weights and conductivities. Then, a positive feedback mechanism is employed to accelerate convergence of the algorithm. Here, the positive feedback mechanism is an interaction between conductivities and resources along each link. A link with few resources leads to a weak conductivities. The weak conductivities produce a further decrease of resources along the link. Similarly, a link with more resources cases stronger conductivities and further contributes itself to obtain more resources. Finally, the resources of a node are the sum of its inlinks’ resources. This process will continue until each nodes’ resources are steady.

Therefore, in this paper, based on the primary LeaderRank, an extended algorithm, called ***Physarum*Spreader**, is proposed for capturing the spreading ability of nodes in weighted networks.

### 2.1 General Flow of *Physarum*Spreader

**Fig 1:**
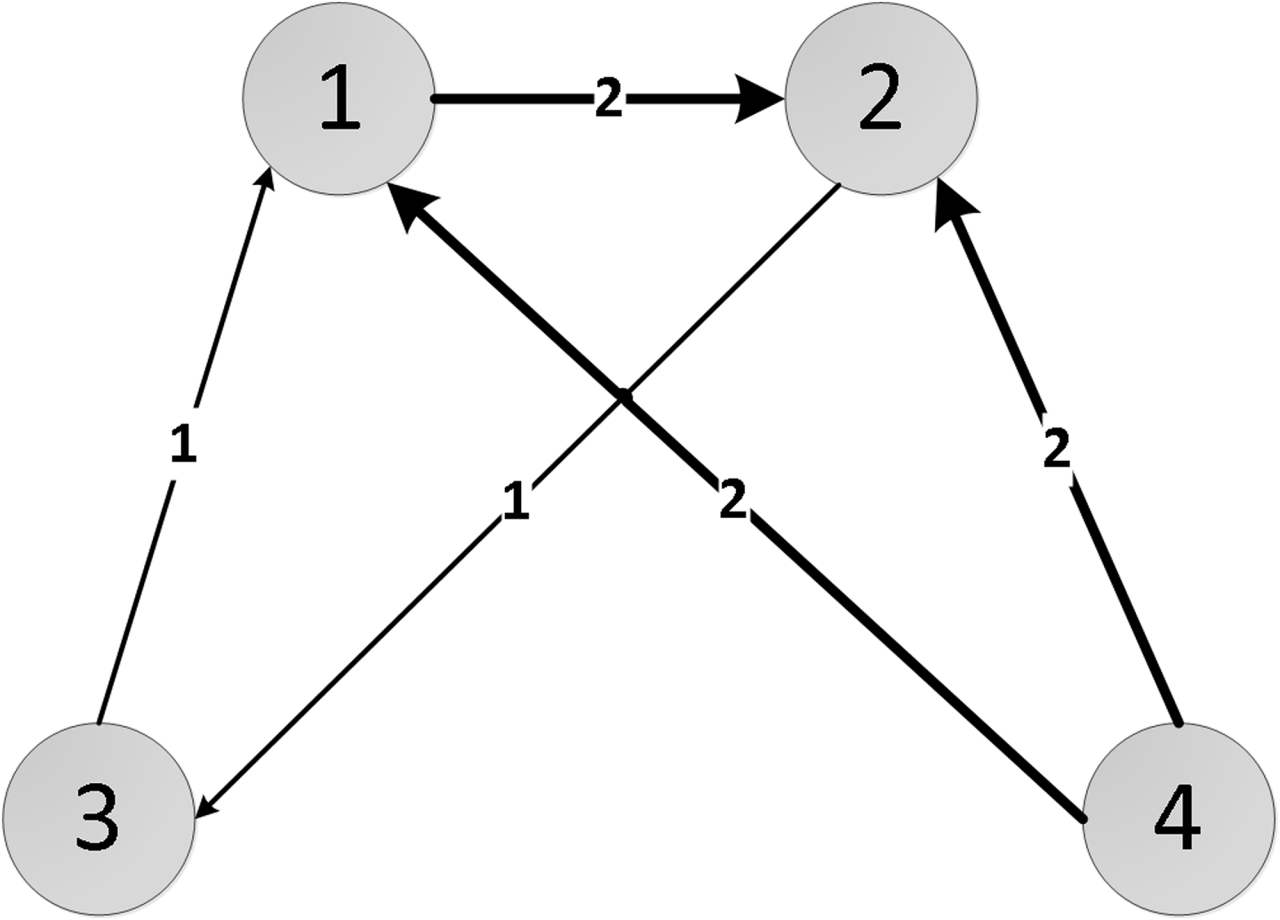
An example network *Net*.

The general flow of our proposed *Physarum*Spreader is described as follows, along with graphical demonstration of a random directed example network *Net* as shown in Fig 1.

**Fig 2:**
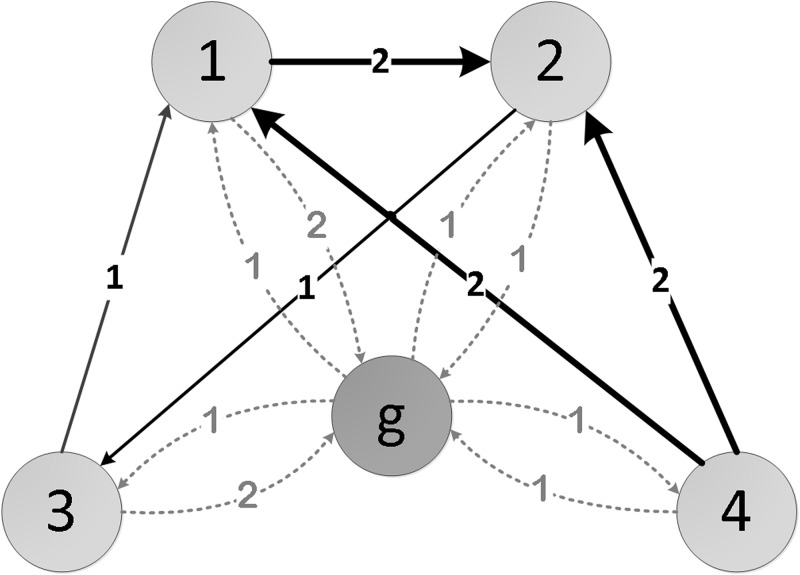
Ground node insertion in a given example network *Net*.

**Fig 3:**
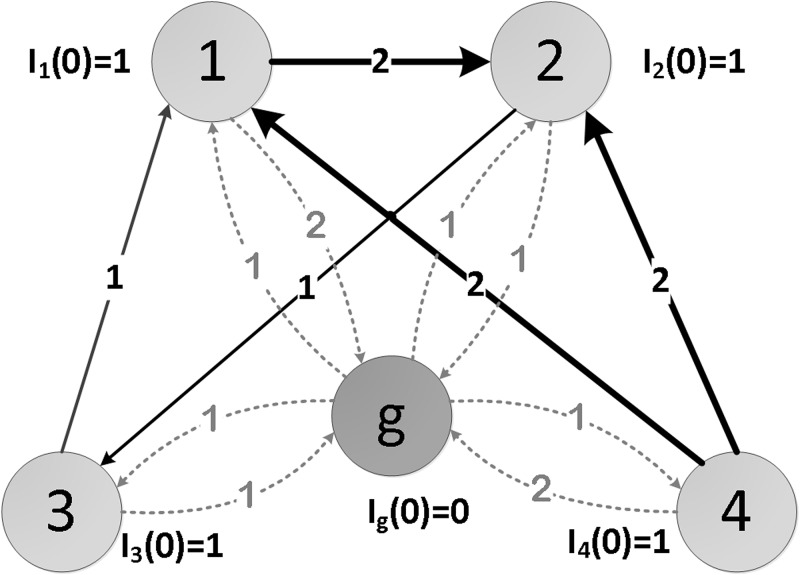
Initialization of flux for nodes in *Net*.

Step 1 Add a **ground node** into the network by connecting every node through bidirectional links (Fig 2). The weight *w*_*ig*_ of the inlink which direction is form node *i*(*i* ∈ *N*) to ground node is determined by the following equation:
  $$\begin{array}{c} w_{ig}=\left\{\begin{array}{cc} \frac{\sum_{j=1}^{l_{i}^{out}}w_{ij}}{l_{i}^{out}}, & \;\;if\;\;\;l_{i}^{out}>0\\ 1, & \;\;if\;\;\;l_{i}^{out}=0 \end{array}\right. \end{array}$$ (7)
  where $l_{i}^{out}$ denotes the total number of outlinks from node *i* without considering weight and node *j* represents the neighbour of node *i*. If the network is directed, this step guarantees the network to be strongly connected.
Step 2 Initialize all nodes (other than the ground node) with unit of resource and the ground node with a score of 0 (Fig 3).
  $$\begin{array}{c} \begin{array}{c} I_{g}\left(0\right)=0,\;where\;g\;is\;ground\;node\\ I_{i}\left(0\right)=1,\;where\;i\in \left\{1,2,\cdots ,N+1\right\}\backslash \left\{g\right\} \end{array} \end{array}$$ (8)
Step 3 Distribute each node’s flux to its neighbors through the out-going edges according to their edge weights.
  $$\begin{array}{c} Q_{ij}\left(t\right)=\frac{w_{ij}D_{ij}\left(t\right)}{\sum_{j=1}^{N+1}w_{ij}D_{ij}\left(t\right)} \end{array}$$ (9)
  where *D*_*ij*_ is the conductivity of each edge with initial value as 1. It is notable that the value of *w*_*ij*_ varies under different circumstances. If the given network is binary, *w*_*ij*_ = 1 for all edges in the network. If the network is weighted and the weight refers to the cost of traversing the edge, *w*_*ij*_ will be the reciprocal of the edge weight. But if the weight stands for the strength of edge relation, such as the number of social proximities, *w*_*ij*_ will be assigned as the weight.
  The initial flux distribution on each out-going edge of nodes at time *t* = 0 is calculated, shown in matrix Q(0):
  $$\begin{array}{c} Q\left(0\right)=\left[\begin{array}{lllll} 0 & 0.5 & 0 & 0 & 0.5\\ 0 & 0 & 0.5 & 0 & 0.5\\ 0.5 & 0 & 0 & 0 & 0.5\\ 0.33 & 0.33 & 0 & 0 & 0.33\\ 0.25 & 0.25 & 0.25 & 0.25 & 0 \end{array}\right] \end{array}$$
Step 4 Adapt the conductivity according the flux through each edge using the following equation:
  $$\begin{array}{c} D_{ij}\left(t+1\right)=\left[Q_{ij}\left(t\right)+D_{ij}\left(t\right)\right]/2,\;where\;\;i,j\neq g \end{array}$$ (10)
  The edge conductivity at time *t* = 1 can be calculated by Eq 10, according to the flux distribution of time *t* = 0. The result is shown in matrix D(1):
  $$\begin{array}{c} D\left(1\right)=\left[\begin{array}{lllll} 0 & 0.75 & 0 & 0 & 1\\ 0 & 0 & 0.75 & 0 & 1\\ 0.75 & 0 & 0 & 0 & 1\\ 0.67 & 0.67 & 0 & 0 & 1\\ 1 & 1 & 1 & 1 & 1 \end{array}\right] \end{array}$$
Step 5 Update the resources of each node for the next iteration, according to the flux flowing into the node and its current score.
  $$\begin{array}{c} I_{i}\left(t+1\right)=\sum_{j=1}^{N+1}Q_{ji}\left(t\right)I_{j}\left(t\right) \end{array}$$ (11)
Step 6 Determine whether the steady state of nodes’ score is attained. If it converges to a steady state, the flux of the ground node is evenly distributed to all other nodes. And the final score *S*_*i*_ for each node’s spreading performance is attainted as:
  $$\begin{array}{c} S_{i}=I_{i}\left(t_{c}\right)+\frac{I_{g}\left(t_{c}\right)}{N},\;where\;i\in \left\{1,2,\cdots ,N\right\} \end{array}$$ (12)
  If the state is not steady yet, the process continues to Step 3.

The final resources for each node in *Net* is: *S*_1_(1.0100) > *S*_2_(1.0097) > *S*_3_(1.0002) > *S*_4_(0.9801).

## 3 Comparisons and Tests

In this section, the proposed method is compared with another four approaches (degree, betweenness, k-shell, weighted PageRank) to demonstrate its effectiveness. The four methods will be defined in section 3.1. What’s more, all methods are tested on noisy data to evaluate their stability. Two real networks are employed. One of them is a directed and weighted network. It is the network of the 500 busiest commercial airports in the United States. A tie exists between two airports if a flight was scheduled between them in 2002. The weights correspond to the number of seats available on the scheduled flights [54]. You can obtain the data through the hyperlink listed in S1 Text. The other one is an undirected and weighted network reorganized by Newman [55–57]. It is a collaboration network of scientists posting preprints on the high-energy theory archive at www.arxiv.org. These papers appeared in a 5-year window, from 1995 to 1999 inclusive. The data can also be downloaded through the S1 Text. More attributes about the two networks are listed in Table 1.

**Table 1:** Basic statistics of the two real networks.

Networks	Nodes	Edges	Diameter	Average Clustering Coefficient	Average Degeree
US airports network	1572	28235	9	0.469	17.961
collaborations network	8361	15751	19 7.025	0.636	3.768

### 3.1 Definitions of the compared centrality measures

In the context of social science, the topology of a social network is represented by an adjacency matrix *A* = {*w*_*ij*_}_*N*×*N*_, where the element *w*_*ij*_ > 0 if there exists a link from *j* to *i* and *w*_*ij*_ = 0 otherwise. For an undirected network, A is a symmetric matrix with *w*_*ij*_ = *w*_*ji*_. If the network is weighted, the element *w*_*ij*_ represents the weight of the link from *j* to *i*. Actually, the adjacency matrix *A* fully describes the topological structure of the social network. Here, the adopted centrality measures to make comparisons are calculated by the following equations:

(1)Degree *k*_*i*_ for a node *i* can be computed as follows:

$$\begin{array}{c} k_{i}=\sum_{j=1}^{N}w_{ij}+\sum_{j=1}^{N}w_{ji} \end{array}$$ (13)

(2)Betweenness *C*_*B*_(*i*) is defined as

$$\begin{array}{c} C_{B}\left(i\right)=\sum_{s\neq i\neq t}\frac{\sigma_{st}\left(i\right)}{\sigma_{st}} \end{array}$$ (14)

where *σ*_*s*_ *t* is the number of the shortest paths between nodes *s* and *t*, and *σ*_*st*_(*i*) is the number of the shortest paths between *s* and *t* which pass through node *i*.

(3)K-shell [58]: The k-shell index of a node is obtained by a procedure called *k*-shell decomposition, where we successively prune nodes in the network layer by layer. Concretely, the decomposition starts by removing nodes with degree *k* = 1. After that, some nodes may have only one link left. So we continue pruning the network iteratively until there are no nodes with *k* = 1. The removed nodes fall into a *k*-shell with index *k*_*S*_ = 1. With the similar method, we iteratively remove the next *k* shell *k*_*S*_ = 2 and higher *k* shells until all nodes are pruned. In the decomposition procedure, each node is assigned with a *k*-shell index. The periphery of the network corresponds to small *k*_*S*_ and the nodes with high *k*_*S*_ define the core of the network.

(4)Weighted PageRank [59] can be calculated from:

$$\begin{array}{c} PR_{i}\left(t\right)=\alpha \sum_{j=1}^{N}\frac{w_{ij}PR_{j}\left(t\right)}{k_{j}^{out}}+\frac{1-\alpha }{N} \end{array}$$ (15)

where $k_{j}^{out}=\sum_{i=1}^{N}w_{ji}$ and *α* is the jumping probability. *PR*_*i*_(*t*) is the probability that node *i* is visited by the random walker at time *t*. As time *t* increases, the probability *PR*_*i*_(*t*) will converge to a stationary probability *PR*_*i*_. This value is defined as the PageRank which are used to determine its ranking relative to other nodes. In the calculation, the conventional choice of *α* is 0.85. In this paper, *α* is set as 0.85 for all experiments.

### 3.2 Effectiveness

A modified susceptible-infected-removed (SIR) model is employed to estimate the spreading influence of the top-ranked nodes in weighted networks. In this model, individuals can be in three discrete states: susceptible, infected or removed. Each individual in the model can be represented by a node of the network and can only spread infection to its neighbors along the outgoing edges in the network. At each step, each infected node *i* randomly chooses one of its susceptible neighbors, *j*, and infects it with probability *λ*_*ij*_, and then be removed (dead or recovered with immunity) with probability *β*. The probability *λ*_*ij*_ is determined by the following equation [60]:

$$\begin{array}{c} \lambda_{ij}={\left(\frac{w_{ij}}{w_{max}}\right)}^{\alpha },\;\alpha >0 \end{array}$$

where *α* is a positive constant and *w*_*max*_ is the largest value of *w*_*ij*_ in the network. Since $\frac{w_{ij}}{w_{max}}<1$, the smaller the *α* is, the more quickly the infection spreads. The process stops when no infected node is present. Here we use the cumulative number of infected nodes (which includes infected and recover nodes), denoted by *N*, as a function time. Without the loss of generality, *α* and *β* are assigned as 0.2 and 1.

We first compare the spreading processed activated by top-ranked [61] nodes from *Physarum*Spreader and another four centrality measures (degree, betweenness, k-shell, weighted PageRank). Taking weighted PageRank as an example. If among two top-*L* lists by *Physarum*Spreader and weighted PageRank, there are *n* different nodes, we compare the spreading processed activated by these *n* different nodes, respectively. For example, if *L* = 5, the top-ranked lists are {97, 1016, 754, 333, 335} and {754, 1222, 97, 841, 1016} for weighted PageRank and *Physarum*Spreader, then *n* = 3 and the spreading processes are compared with initially infected nodes {333, 335} for weighted PageRank and {1222, 841} for *Physarum*Spreader.

Tables 2 and 3 list the top 50 nodes obtained by different centrality methods in US airports network and collaborations network.

**Table 2:** Top 50 nodes ranked by different centrality methods for US airports network.

Rank	Degree	Betweenness	K-shell	Weighted PageRank	*Physarum*Spreader
1	97	76	27	97	754
2	1016	584	65	1016	1222
3	604	1331	97	754	97
4	654	1433	106	333	841
5	754	207	126	335	1016
6	422	591	148	654	774
7	333	604	176	1219	1219
8	907	428	181	76	76
9	606	892	184	606	333
10	335	1520	206	1222	654
11	866	126	207	866	1544
12	368	1542	212	752	790
13	1062	1129	261	1064	33
14	752	654	267	907	325
15	184	343	271	267	582
16	841	1557	306	422	599
17	445	1006	325	841	985
18	1222	427	328	368	207
19	267	686	333	1062	1457
20	1219	136	335	184	866
21	207	431	368	604	132
22	261	60	405	1250	241
23	212	754	422	445	427
24	176	1238	445	573	335
25	850	166	497	774	907
26	1557	206	582	212	606
27	1064	866	604	427	573
28	1072	722	606	1190	1250
29	1250	1051	624	1050	136
30	998	93	649	328	264
31	584	1287	654	132	1006
32	870	1032	752	846	267
33	1148	1566	754	1369	1064
34	838	1263	774	790	60
35	328	573	787	1287	422
36	774	107	838	779	368
37	624	394	841	850	1029
38	1287	365	846	1557	1426
39	271	1125	850	1457	78
40	106	466	863	1244	492
41	910	1294	866	985	184
42	846	422	870	968	661
43	1212	907	907	584	39
44	614	498	910	838	604
45	1192	184	985	870	968
46	148	670	998	261	445
47	76	752	1005	1258	1062
48	306	710	1008	1239	752
49	1369	503	1016	1263	212
50	126	36	1017	1148	342

**Table 3:** Top 50 nodes ranked by different centrality methods for collaborations network.

Rank	Degree	Betweenness	K-shell	Weighted PageRank	*Physarum*Spreader
1	87	1571	6790	24	24
2	480	1832	6791	87	1794
3	168	24	6792	763	547
4	24	975	6793	997	546
5	997	3473	6794	546	473
6	481	500	6795	530	415
7	656	1603	6796	547	763
8	926	1368	6797	480	474
9	1571	926	6798	168	106
10	546	87	6799	469	926
11	975	1617	6800	433	123
12	473	276	6801	1424	2855
13	530	1337	6802	1201	87
14	547	481	6803	1794	480
15	884	193	6804	675	1340
16	930	930	6805	816	469
17	1603	2544	6806	975	39
18	39	480	6807	926	124
19	123	6487	6808	2117	1452
20	956	387	6809	106	479
21	106	168	6810	415	3940
22	675	752	6811	164	1087
23	1171	165	6812	2254	197
24	1201	307	6813	2855	38
25	1516	4201	310	177	420
26	2117	1190	5687	1941	3402
27	38	5248	5688	1698	1548
28	310	502	5689	884	431
29	763	1738	5690	1754	1754
30	1393	2286	5691	896	195
31	2070	5908	5692	1528	1641
32	1286	4490	5693	1479	37
33	185	1275	5694	473	1511
34	193	1515	5695	474	1870
35	431	6505	5696	1444	1512
36	433	3140	5697	336	178
37	469	997	5698	38	517
38	4201	185	5699	41	1571
39	221	890	5700	1736	1642
40	449	166	5701	1452	518
41	1126	1393	5702	39	530
42	1255	1479	5703	23	322
43	1479	3770	5704	1854	2116
44	1570	404	53	85	2217
45	1698	501	530	956	3717
46	1754	948	546	616	321
47	35	1058	547	631	1640
48	41	956	1831	592	433
49	371	103	4836	1241	956
50	474	1247	5248	420	168

**Fig 4:**
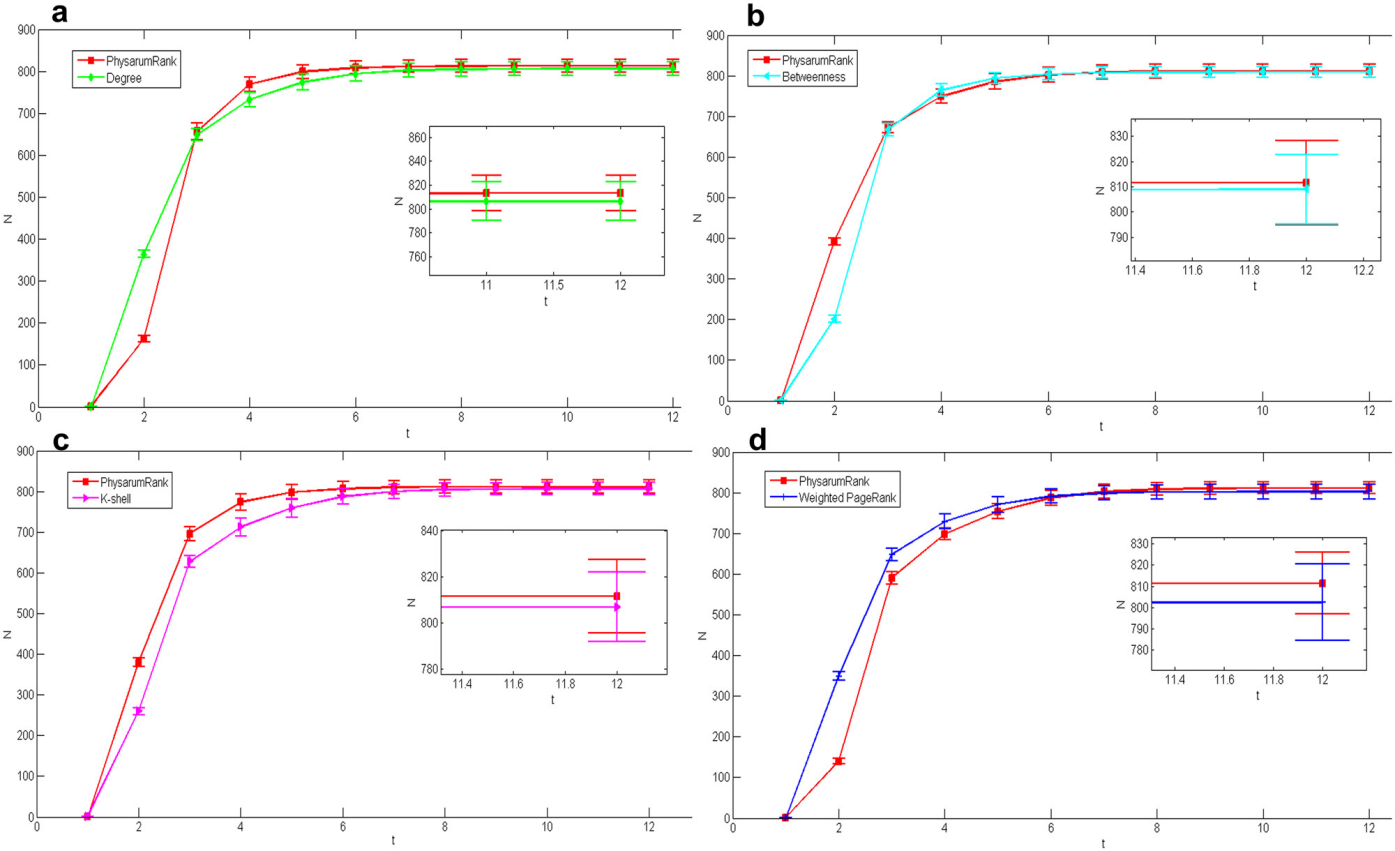
Comparison of epidemic spreading efficiency among different measures when *L* = 20, in US airports network. Each result *N* is obtained by averaging over 100 implementations with *α* = 0.2 and *β* = 1. (a):*Physarum*Spreader vs Degree, *n* = 11. (b):*Physarum*Spreader vs Betweenness, *n* = 17. (c):*Physarum*Spreader vs K-shell, *n* = 16. (d):*Physarum*Spreader vs Weighted PageRank, *n* = 10.

**Fig 5:**
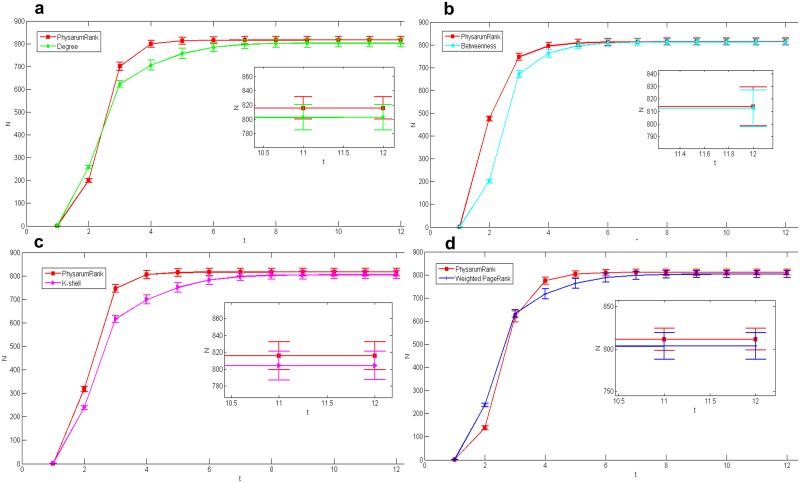
Comparison of epidemic spreading efficiency among different measures when *L* = 50, in US airports network. Each result *N* is obtained by averaging over 100 implementations with *α* = 0.2 and *β* = 1. (a):*Physarum*Spreader vs Degree, *n* = 24. (b):*Physarum*Spreader vs Betweenness, *n* = 35. (c):*Physarum*Spreader vs K-shell, *n* = 27. (d):*Physarum*Spreader vs Weighted PageRank, *n* = 18.

In US airports network, Fig 4a, 4b, 4c and 4d show the spreading results compared with degree centrality, betweenness, k-shell and weighted PageRank corresponding to *n* = 11, 17, 16 and 10, respectively, when *L* equals to 20. As can be seen, the proposed method slightly outperforms the other four centrality measures. In order to verify the efficacy of the proposed method, we check its efficiency among more nodes. Since it is impossible to check all nodes, we selected the 50 most important nodes to conduct the experiment. Fig 5a, 5b, 5c and 5d display the spreading results corresponding to *n* = 24, 35, 27 and 18 under *L* = 50. Here we can see, the proposed algorithm exhibits a good efficiency in *L* = 50 as well as in *L* = 20.

**Fig 6:**
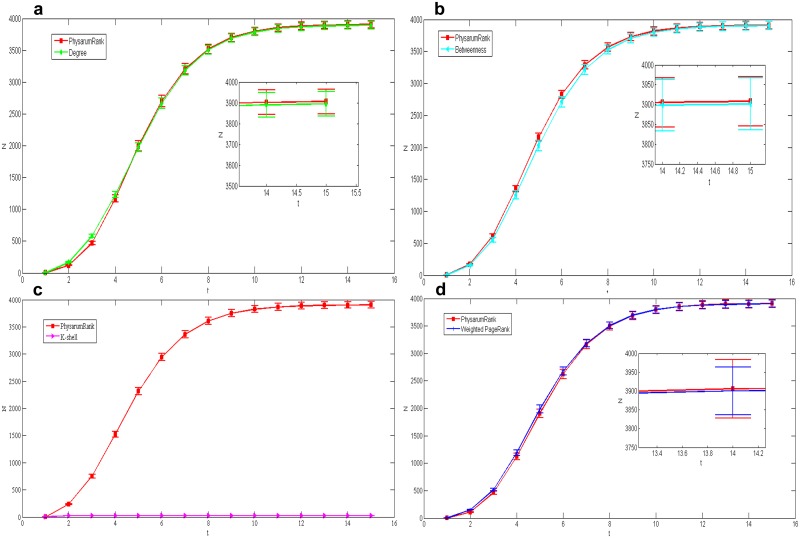
Comparison of epidemic spreading efficiency among different measures when *L* = 20, in collaborations network. Each result *N* is obtained by averaging over 100 implementations with *α* = 0.2 and *β* = 1. (a):*Physarum*Spreader vs Degree, *n* = 11. (b):*Physarum*Spreader vs Betweenness, *n* = 16. (c):*Physarum*Spreader vs K-shell, *n* = 20. (d):*Physarum*Spreader vs Weighted PageRank, *n* = 10.

**Fig 7:**
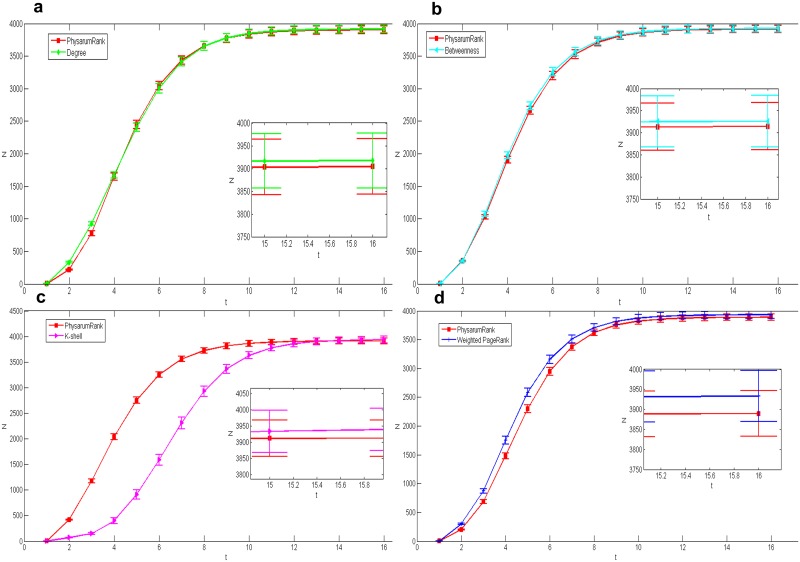
Comparison of epidemic spreading efficiency among different measures when *L* = 50, in collaborations network network. Each result *N* is obtained by averaging over 100 implementations with *α* = 0.2 and *β* = 1. (a):*Physarum*Spreader vs Degree, *n* = 29. (b):*Physarum*Spreader vs Betweenness, *n* = 43. (c):*Physarum*Spreader vs K-shell, *n* = 47. (d):*Physarum*Spreader vs Weighted PageRank, *n* = 27.

We also applied our algorithm in the other network with bigger size: collaborations network. Fig 6a, 6b, 6c and 6d show the spreading results compared with another four centrality measures corresponding to *n* = 11, 16, 20 and 10, respectively, under *L* = 20. The figures show that the cumulative number of the infected nodes obtained by our method is a bit bigger than the results calculated by the others except k-shell. However, when *L* equals to 50 in Fig 7, our method is a little bit worse than other centrality measures but has a quicker spread velocity than k-shell while *n* = 29, 43, 47 and 27.

With *L* increasing, more and more important nodes overlap, those overlapped nodes will be removed from the top-ranked lists. It means that the rest different nodes may have less spreading influence than those removed. Thus, the cumulative numbers of infected nodes have little changes in spite of *n* and *L* increasing.

### 3.3 Robustness

To scientifically test the performance of the *Physarum*Spreader algorithm, we measure the change in rankings when links are randomly removed with probability form 0.1 to 0.5. The rankings obtained from the modified network are compared to those from the original network, by measuring the impact *I*_*R*_ on ranking, as given by [9]

$$\begin{array}{c} I_{R}=\sum_{i=1}^{N}|R_{i}^{{}^{\prime }}-R_{i}| \end{array}$$ (16)

3 and $R_{i}^{{}^{\prime }}$ correspond to the rankings obtained respectively from the original and modified graph. We measure *I*_*R*_ for *Physarum*Spreader, weighted PageRank, degree, betweenness and k-shell subject to the same modifications.

**Fig 8:**
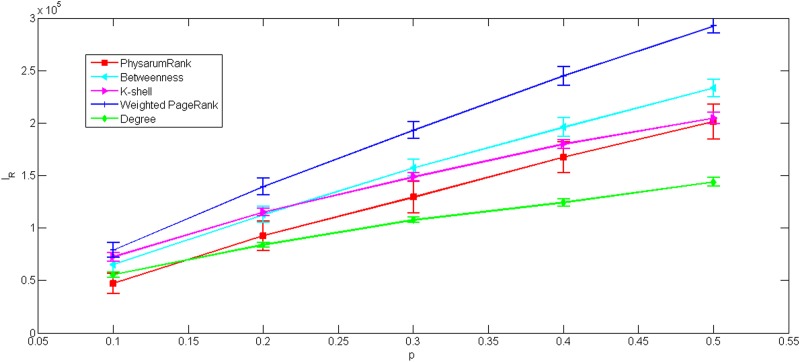
The impact ranking as a function of number of links randomly removed for US airports network. All data points are average values over 100 independent runs with error bars showing the standard deviations.

**Fig 9:**
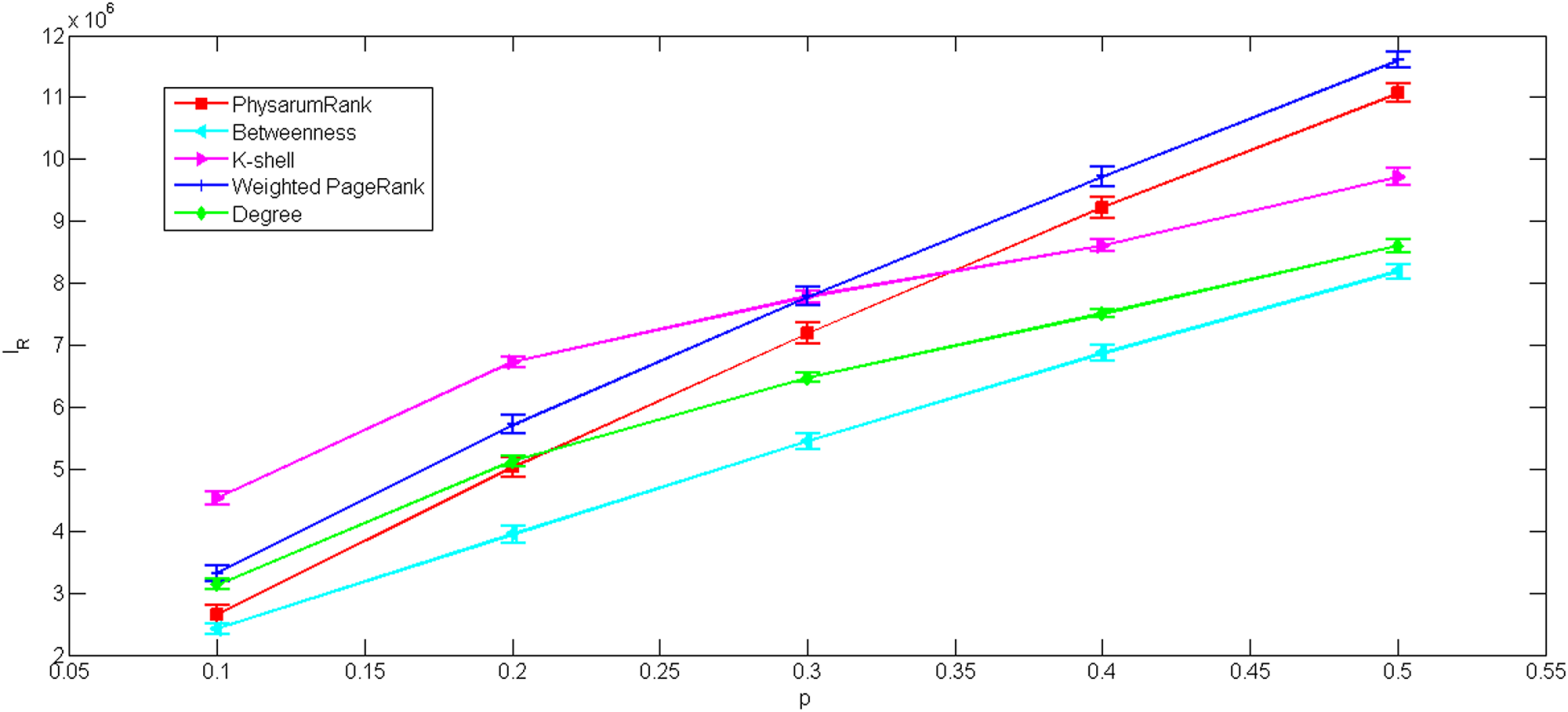
The impact ranking as a function of number of links randomly removed for collaborations network. All data points are average values over 100 independent runs with error bars showing the standard deviations.

As shown in Figs 8 and 9, *I*_*R*_ increase with the number of links removed. In Fig 8, we can observe that our method is obviously more tolerant than weighted PageRank, betweenness and k-shell but worse than degree.

Here we can see, robustness of the presented approach is also better than weighted PageRank, in Fig 9. However, when *p* > 0.2, the tolerance of our method underperform degree and is a little worse than k-shell while *p* > 0.3. Notably, it seems that betweenness becomes the most tolerant method, but it’s not true. Due to the topology of the original network, there are 4568 nodes whose value of betweenness equal to 0. It means that the rankings of these nodes remain unchanged when the network is modified. Hence, the tolerance of betweenness is evaluated invalidly, when it is tested in collaborations network by this measure.

In practical application, a valuable ranking algorithm should not be effective only, but also be robust. To further demonstrate the proposed method’s advantages in terms of robustness, we demonstrate *Physarum*Spreader algorithm in a representative example called *sybil attack*[62]. Consider a situation that spammers deliberately gain disproportionately high rank by creating huge fake entities. To simulate this attack, each time node *i* creates *v*(*v* = 10, 50, 100) fake entities which only direct to node *i* with weight equaling to 1. The rank of node *i* is denoted by *r*_*i*_ first and then it is represented by $r_{i}^{{}^{\prime }}$ after manipulations. Obviously, $r_{i}^{{}^{\prime }}$ is less than or equal to *r*_*i*_ and if there are smaller differences between them, the approach will be more robust. Only the top-100 users (*i* = 1, 2, 3, …, 100) are studied under this attack.

**Fig 10:**
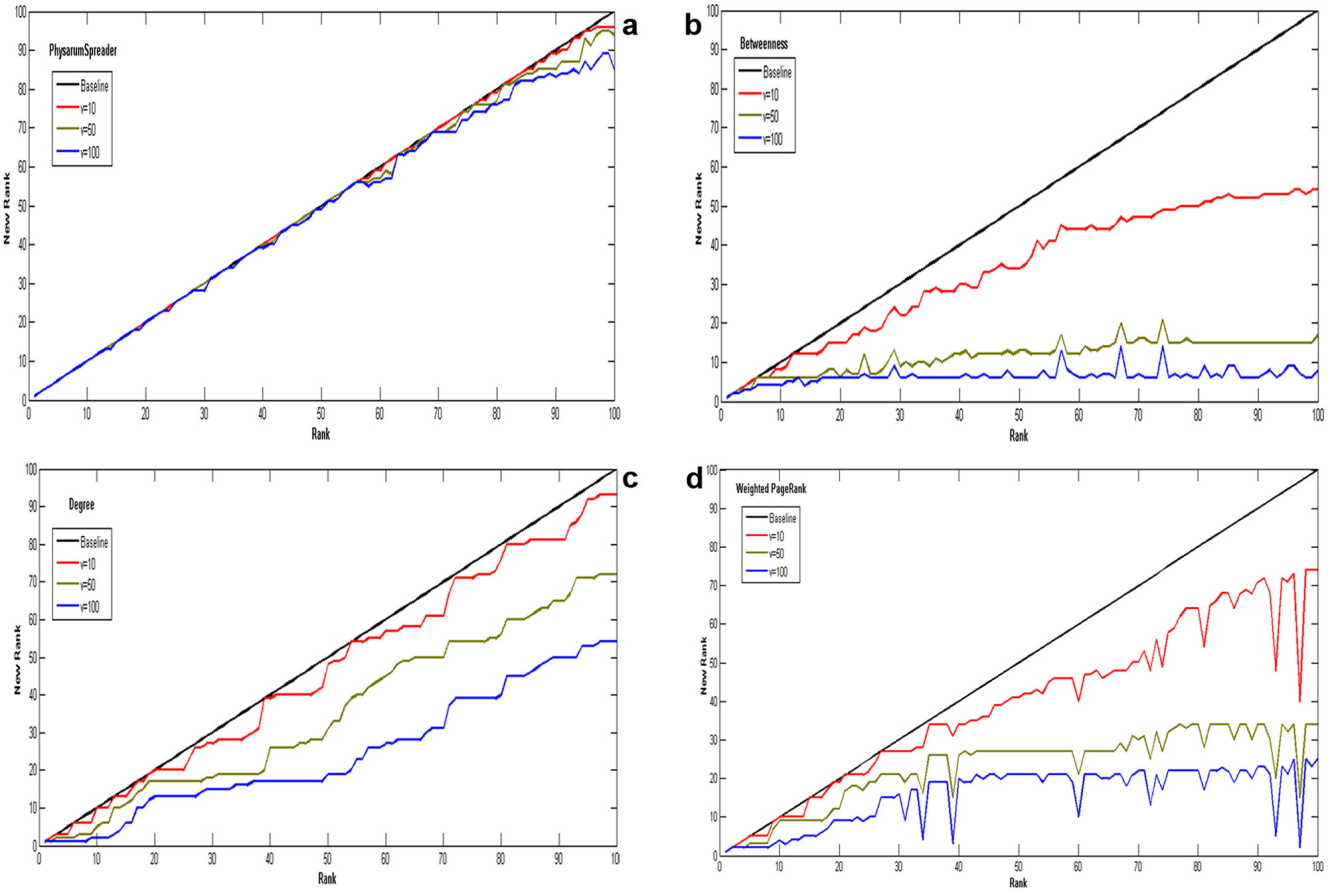
The impact ranking as a function of number of links randomly removed for US airports network. All data points are average values over 100 independent runs with error bars showing the standard deviations.

**Fig 11:**
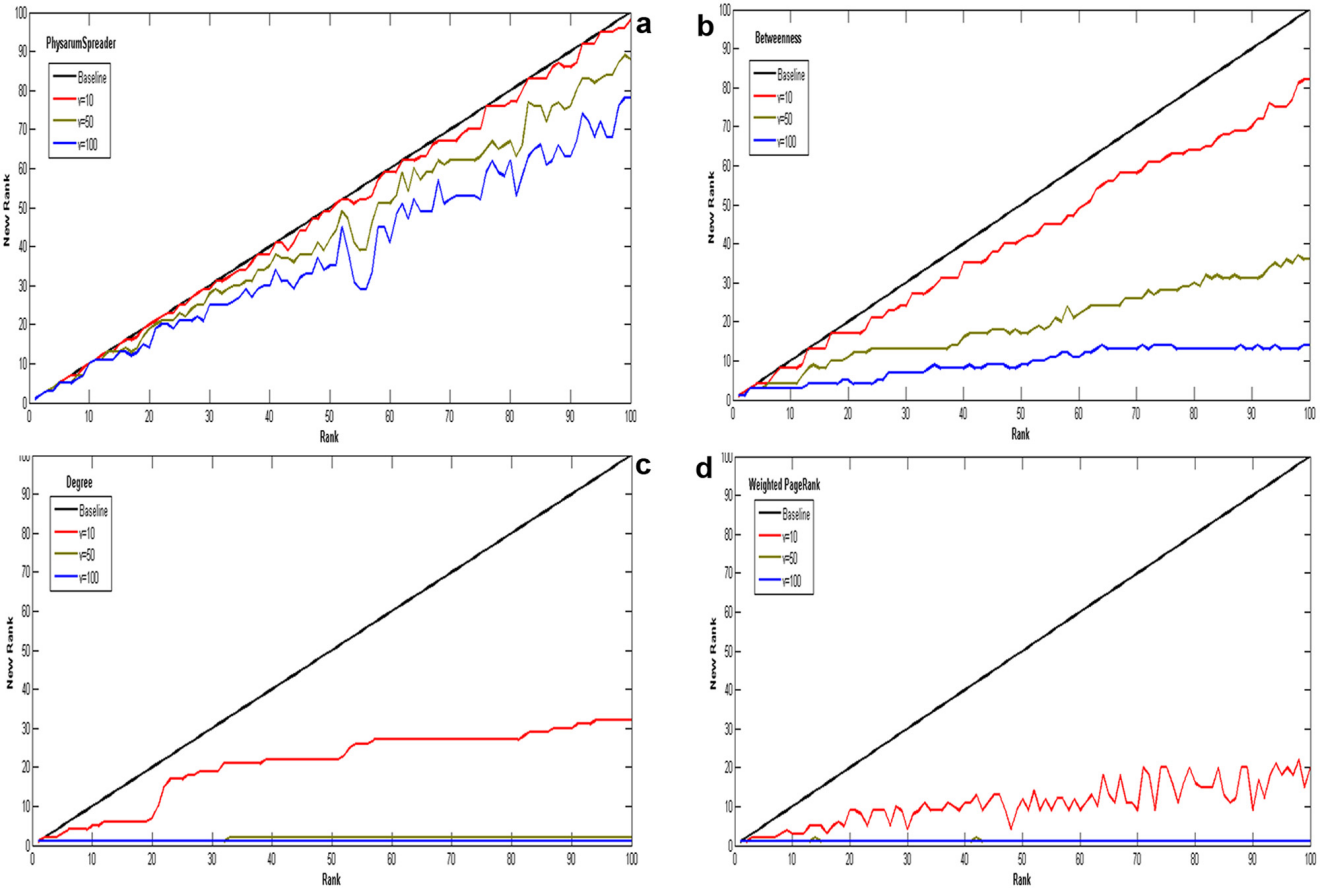
The impact ranking as a function of number of links randomly removed for collaborations network. All data points are average values over 100 independent runs with error bars showing the standard deviations.

As shown in Figs 10 and 11, the vertical axis displays the manipulated rank of a user after creation of *v* fake fans and the horizontal axis shows its original rank. As we can see, in both networks of US airports network and collaborations network, *Physarum*Spreader is the most robust algorithm among others against manipulations as its smaller change of rank.

## 4 Conclusions

In this paper, focus is placed on identifying influential spreaders in weighted networks and an extended algorithm called *Physarum*Spreader has been proposed based on LeaderRank and a positive feedback mechanism inspired from *Physarum Polycephalum*. In order to investigate the performance of the proposed method, two weighted real networks that one is directed and the other is undirected, have been used as test network data sets. Furthermore, comparison with four well-known centrality measures are also studied with the help of an epidemic spreading model. Experimental results indicate that *Physarum*Spreader is effective in identifying influential spreaders. In addition, the proposed method has a good robustness compared with other measures.

## Supporting Information

S1 Text Dataset web pages. (TXT)
